# Chronic Intranasal Treatment with an Anti-Aβ_30-42_ scFv Antibody Ameliorates Amyloid Pathology in a Transgenic Mouse Model of Alzheimer's Disease

**DOI:** 10.1371/journal.pone.0018296

**Published:** 2011-04-05

**Authors:** Susann Cattepoel, Michael Hanenberg, Luka Kulic, Roger M. Nitsch

**Affiliations:** Division of Psychiatry Research, University of Zurich, Zurich, Switzerland; University of California, Los Angeles, and Cedars-Sinai Medical Center, United States of America

## Abstract

Amyloid-beta peptide (Aβ)-directed active and passive immunization therapeutic strategies reduce brain levels of Aβ, decrease the severity of beta-amyloid plaque pathology and reverse cognitive deficits in mouse models of Alzheimer's disease (AD). As an alternative approach to passive immunization with full IgG molecules, single-chain variable fragment (scFv) antibodies can modulate or neutralize Aβ-related neurotoxicity and inhibit its aggregation *in vitro*. In this study, we characterized a scFv derived from a full IgG antibody raised against the C-terminus of Aβ, and studied its passage into the brains of APP transgenic mice, as well as its potential to reduce Aβ-related pathology. We found that the scFv entered the brain after intranasal application, and that it bound to beta-amyloid plaques in the cortex and hippocampus of APP transgenic mice. Moreover, the scFv inhibited Aβ fibril formation and Aβ-mediated neurotoxicity *in vitro*. In a preventative therapeutic approach chronic intranasal treatment with scFv reduced congophilic amyloid angiopathy (CAA) and beta-amyloid plaque numbers in the cortex of APPswe/PS1dE9 mice. This reduction of CAA and plaque pathology was associated with a redistribution of brain Aβ from the insoluble fraction to the soluble peptide pool. Due to their lack of the effector domain of full IgG, scFv may represent an alternative tool for the treatment of Aβ-related pathology without triggering Fc-mediated effector functions. Additionally, our observations support the possibility that Aβ-directed immunotherapy can reduce Aβ deposition in brain vessels in transgenic mice.

## Introduction

Alzheimer's disease (AD) is the most common form of dementia in the aged population and the number of patients is continuously increasing due to a lack of effective treatments. Therefore, considerable efforts focus on the development of efficacious therapies [1]. AD is characterized by progressive memory deficits and cognitive impairments. The histopathological characteristics are extracellular amyloid deposits and intracellular neurofibrillary tangles. The accumulation of these protein aggregates is accompanied by synaptic dysfunction, inflammation and eventually neuronal death [2]. Generation of the amyloid β- peptide (Aβ) from the amyloid precursor protein (APP) by β- and γ-cleavage followed by seeded aggregation of Aβ is widely believed to be the initiating event in the pathogenesis of AD [3] resulting in sustained deposition of Aβ in brain parenchyma and cerebral blood vessels [4]. Because Aβ aggregates are neurotoxic, numerous strategies to prevent Aβ aggregation and accumulation are currently studied as potential ways to treat or prevent AD.

Aβ immunotherapy was first introduced by Schenk et al. [5], who demonstrated that vaccination with Aβ_1-42_ and Freund's adjuvant not only prevented Aβ accumulation in younger PDAPP mice but also cleared pre-existing amyloid plaques in older animals. This first study was followed by several preclinical studies in different transgenic AD mouse models, which substantiated the therapeutic potential of targeting Aβ by active immunization or passive administration of anti-Aβ antibodies [5], [6]. In view of these promising results, animal experiments were soon followed by a human clinical trial with aggregated Aβ_1-42_ and the QS-21 adjuvant (Elan/Wyeth AN1792 trial). The Phase I clinical trial demonstrated apparent safety and tolerability of AN1792, which elicited a significant antibody response to Aβ_1-42_ [7]. A subsequent Phase II clinical trial was halted due to aseptic meningoencephalitis in 6% of the treated patients [8]. A long-term follow-up study of the Zurich cohort revealed increased titers of Aβ-specific antibodies [9], [10] and a slowed cognitive decline in patients who had responded to the active vaccine as compared to non-responders [11]. The cases of meningoencephalitis did not correlate with the presence or concentration of antibodies to Aβ [8], [9], and it is now generally believed that the meningoencephalitis was due to a T-cell response against Aβ [2], [12]. Histopathological analysis of brain tissue obtained from individual patients who had died from unrelated causes revealed beta-amyloid plaque clearance in association with increased antibody titers, with some brain regions virtually free of beta-amyloid plaques [1].

Because of the adverse side effects of active vaccination, passive immunization with humanized monoclonal antibodies against Aβ is vigorously pursued as an alternative approach [13], [14]. In several studies it was shown that this therapeutic approach was rather successful in ameliorating amyloid pathology but that it was not free of side effects either. Intracerebral hemorrhages associated with cerebral amyloid angiopathy (CAA) were increased in APP transgenic mice treated with some antibodies, especially those binding to amyloid plaques [15], [16], [17]. Antibody-mediated microhemorrhages may be related to interactions of anti-Aβ antibodies with vascular amyloid causing structural fragility of degenerated vessel walls [17]. Another possible explanation might be related to Fc effector functions since de-glycosylated IgG without effector function were still effective in clearing amyloid plaques, but had a significantly reduced ability to induce microhemorrhages in transgenic mice [18]. Additionally, it was shown that antibody therapeutics can lead to increased inflammatory responses mediated by the Fc domain of full IgG antibodies. Moreover, Bacskai et al. reported that F(ab)_2_ fragments were also capable of removing plaques providing evidence that non-Fc-mediated mechanisms may be involved in the clearance of beta-amyloid by passive immunotherapy [19].

In view of these findings, single chain variable fragments (scFv) of IgG, lacking the Fc effector domain, have evolved as a novel and promising therapeutic tool for the treatment of AD. ScFvs have a low molecular weight of less than 30 kDa which might facilitate their transfer across the blood-brain barrier into the brain. ScFvs consist of a single polypeptide chain, comprising an antibody heavy chain variable domain (VH) associated by a flexible polypeptide linker to a light chain variable domain (VL) [20], [21], [22]. This construct can easily be expressed in E. coli [23], rendering scFv production efficient and cost-effective. Previous *in vitro* studies demonstrated that scFv were capable of inhibiting Aβ_1-42_ aggregation and preventing Aβ-induced neurotoxicity [24], [25], [26], [27], [28]. In addition, two *in vivo* studies showed that scFv can ameliorate amyloid pathology after stereotaxic injection into the hippocampus and cortex of Tg2576 mice [29] or after intracranial adeno-associated virus-mediated delivery of an anti-pan amyloid-beta scFv in TgCRND8 mice [30].

Building on our previous finding that the full IgG 22C4 directed against the C-terminus of Aβ cleared brain amyloid, we tested whether an antibody fragment derived from 22C4 IgG retained this activity [31], [32]. Therefore we characterized a scFv generated by grafting the complementarity determining regions (CDRs) of the VH and VL domains of the 22C4 IgG into a human scFv framework. The resulting 22C4 scFv was expressed in E. coli and characterized in terms of its binding characteristics, and its potential to inhibit Aβ aggregation and prevent Aβ-induced neurotoxicity. Finally, we treated 8 month old APPswe/PS1dE9 intranasally with 22C4 scFv, the full IgG 22C4 and a vehicle control, to evaluate the *in vivo* efficacy of 22C4 scFv to ameliorate beta-amyloid pathology. As scFv have a very short half-life after systemic application due to glomerular filtration we chose the intranasal application as route of delivery. It is known that peptides enter the brain quickly via the nasal route, thus circumventing the blood-brain barrier and renal excretion [33], [34], [35], although the exact transport routes and mechanisms of this way of delivery are to date not yet fully understood [36], [37],[38],[39],[40].

## Materials and Methods

### Generation and production of 22C4 scFv

22C4 scFv is a single chain antibody (scFv) emanating from the Aβ-specific mouse IgG1 antibody 22C4, which was generated by immunizing mice with Aβ_30-42_. Therefore, both 22C4 IgG and 22C4 scFv are directed against the C-terminus of Aβ.

The V_L_ and the V_H_ domains were cloned as published previously [20], [21], [23]. Briefly, the mRNA was derived from hybridoma cells producing the antibody 22C4. An RT-PCR using the primers described by Burmester and Plückthun was performed to amplify the V_L_ and V_H_ domains. The two domains were assembled by a SOE-PCR (splicing by overlap extension). The amplified scFv fragment was digested by SfiI, cloned into a pTFT74 expression vector and sequenced. The mouse scFv antibody obtained had kept its specificity for Aβ_1-42_. Humanisation of scFv was performed by grafting the CDRs of the V_L_ and V_H_ domains into a human scFv framework leading to the single chain antibody 22C4 scFv. For the control scFv Fw 2.3 random CDRs were grafted into the analogous framework, which in both cases carries a 5xHis-Tag and a FLAG-Tag for purification and detection.

Plasmids encoding 22C4 scFv and the control scFv were introduced into BL21 (DE3) E. coli and expressed as inclusion bodies. Functional single chain antibodies were obtained by refolding from inclusion bodies, dialysis and subsequent purification by gel filtration over a Superdex S75 16/60 column (GE Healthcare) which was connected to the Äkta Basic FPLC System (GE Healthcare). Production of 22C4 scFv and the control scFv was done at ESBATech, Schlieren, Switzerland.

### Determination of mass

The exact mass of 22C4 scFv was determined by electro spray mass spectrometry in collaboration with the Functional Genomics Centre (University of Zurich). 22C4 scFv was purified and measured in 50 % acetonitrile/0.2 % formic acid (pH 2). Mass spectra (neutral mass) were deconvoluted using the MaxEnt1 software.

### Stability assay

The midpoint of denaturation of 22C4 scFv was determined in a thermal stability study measuring the Fourier transform-infrared (FT-IR) spectrum using a Bio-ATR-cell on a Bruker Tensor 27 spectrometer. A temperature ramp ranging from 25 to 95°C with a 22C4 scFv concentration of 5 mg/ml was performed. 22C4 scFv was left to equilibrate at each temperature for 1–2 minutes before the spectrum was measured.

### Analytical size exclusion chromatography

Size Exclusion Chromatography (SEC) was performed to verify the specific binding of 22C4 scFv to Aβ_1-42_, which should elute as a single peak, indicating that Aβ_1-42_ and 22C4 scFv eluted together, whereas no binding would lead to two different peaks in the elution profile. For this purpose FITC- Aβ_1-42_ (Bachem, Bubendorf, Switzerland) and 22C4 scFv or the control scFv were preincubated at equimolar concentrations (1 µM) under different conditions (temperature and time), which all led to the same result. The gel filtration was performed on a Superdex 75 10/300 column (GE Healthcare, Munich, Germany), which was connected to the Äkta Basic FPLC System (GE Healthcare). 150 µl of each assay were injected into the column and eluted with filtered and degassed TBS (pH 7.4). Samples were detected by UV absorbance (495 nm and 215 nm) at a flow rate of 0.75 ml/min.

### BioLayer interferometry

BioLayer Interferometry (BLI) is a label-free technology for measuring biomolecular interactions. It is an optical analytical technique allowing for the analysis of the interference pattern of white light reflected from two surfaces: a layer of immobilized protein on the biosensor tip, and an internal reference layer. A change in the number of molecules bound to the biosensor tip causes a shift in the interference pattern. Only molecules binding to or dissociating from the biosensor tip can shift the pattern and thus, generate a response profile on the Octet Q System (ForteBio Inc., Menlo Park, CA). The assays were measured in a 96-well format, where the biosensor tip was moved from well to well for incubation. For the pre-equilibrium the streptavidin–coated biosensor tip was incubated in PBS (pH 7.4) for 300 s, the tip was then loaded with biotinylated-Aβ_1-42_ (250 nM in PBS, pH 7.4) for 400 s. After washing the tip for 600 s in PBS (pH 7.4), the association step with 22C4 scFv (200 nM, 125 nM, 75 nM, 50 nM in PBS) or 22C4 IgG (50 nM in PBS) was carried out for 2100 s followed by a dissociation step for 2100 s in PBS. The assays were analyzed and fitted with the Octet Software 4.0 (ForteBio Inc.).

### Cell culture binding assay

Human embryonic kidney 293 (HEK293T) cells (LGC Standards – ATCC, Molsheim Cedex, France) were cultured in Dulbecco's minimum essential medium (DMEM) supplemented with 10% FCS, 2 mM L-glutamine, HEPES and penicillin/streptomycin in a humidified atmosphere of 5% CO_2_ at 37°C. The cells were plated on fibronectine-coated (Sigma, St. Louis, MO) four-chamber Culture Slides (BD Biosciences, Bedford, MA) at a density of 10000 cells/well 24 h before transfection with an APP-Citrine expressing construct. Another 24 h later, the cells were incubated for 30 min at 4°C with 6E10 antibody (Covance Inc., Princeton, NJ), 22C4 scFv, 22C4 IgG and corresponding controls (Fw 2.3, 2H6C2; all 1∶100). After the binding assay the cells were washed with TBS (pH 7.4) and fixed with 4% PFA for 15 min at RT. For immunocytochemistry the cells were stained o/n at 4°C with a rabbit anti-His polyclonal antibody (1∶500 Abcam, Cambridge, MA) in case of 22C4 scFv and the control scFv, followed by detection with an anti-rabbit Cy3 antibody (1∶1000). 6E10, 22C4 IgG and 2H6C2 were detected directly with an anti-mouse Cy3 antibody (1∶1000). Slides were incubated with secondary antibody for 2 h at RT. All washing steps were done with TBS/0.05% Triton X-100.

### Preparation of Aβ_1-42_

Recombinant Aβ_1-42_ peptide was obtained as ultra pure HFIP film from rPeptide (Bogart, GA) and reconstituted in HFIP, which was evaporated with a constant stream of nitrogen. The peptide was then again resuspended in HFIP and aliquoted in Protein LoBind Tubes (Eppendorf). Again HFIP was evaporated with nitrogen, the tubes were snap frozen in liquid nitrogen and aliquots were stored at −80°C.

For experimental use the HFIP film of the peptide was dissolved in 10 mM NaOH (pH 12), which keeps Aβ_1-42_ in its monomeric form. After neutralization with an equivalent amount of 10 mM HCl (pH 2) the aggregation process was started.

### Anti-Aβ antibody ELISA and competition ELISA

The binding characteristics of 22C4 scFv and the corresponding full IgG 22C4 were measured by ELISA. For the binding activity assay, the plate was coated with 1 µg/ml recombinant Aβ_1-42_ or synthetic Aβ_1-42_. The antibodies were added at concentrations from 0.001 nM to 10000 nM and incubated on the plate for 1 h at RT. The scFv was detected with a mouse anti-His antibody (1∶500; Qiagen, Hilden, Germany) and an anti-mouse HRP antibody conjugate (1∶1000, GE Healthcare), the full IgG was directly detected with the anti-mouse HRP antibody. The absorbance was measured at 450 nm and 620 nm with a Tecan Sunrise plate reader (Tecan, Männedorf, Switzerland).

For the competition analysis the plate was coated with Aβ_1-42_ as previously described. 10 nM 22C4 scFv were preincubated with increasing concentrations of Aβ_1-42_ or scrambled Aβ_1-42_ (0 nM to 1000 nM) for 1 h at 4°C. The preincubated mixtures were added to the coated ELISA plate for 15 min at RT, after incubation these mixtures were transferred to another Aβ_1-42_ -coated plate and treated identically, to confirm that the binding equilibrium was not disturbed. Both plates were subsequently treated as described for the anti-Aβ antibody ELISA.

### Thioflavin T aggregation assay

Inhibition of Aβ fibril formation by scFv was monitored by Thioflavin T (ThT) fluorescence. Recombinant Aβ_1-42_ (in 10 mM NaOH) was incubated at a concentration of 2.5 µM with 2.5 µM, 1.25 µM or 0.25 µM of 22C4 scFv. The assay was performed with 50 µM ThT, 500 mM NaCl, 0.1 mM HCl and 10 mM sodium phosphate at 25°C. The assay was stirred throughout the entire measurement. The fluorescence of four parallel reactions was measured in a Fluorescence Spectrophotometer (Varian Inc.) at 456.2 nm (Excitation) and 489.4 nm (Emission), every 2 min for 180 min. The assay was performed 3 times.

### Cell culture and toxicity studies

For primary rat cortical neuron cultures embryos (E18) were isolated and brains dissected on ice-cold PBS (pH 7.4)/glucose (1 mg/ml) according to standard procedures but with minor changes to the protocol. Briefly, brains were dissected, cortices removed and collected in ice-cold PBS/glucose. Cortices were then stripped off the meninges, minced and incubated in Dispase II (Roche, Indianapolis, IN; 2.4 U/ml) at 37°C for 10 min. The tissue was washed with DMEM plus 10%FCS, triturated and the number of living cells was determined after staining with Trypan Blue. After centrifugation of the cells for 5 min at 1000 rpm and removal of the DMEM/FCS, cells were resuspended and diluted to the desired density in Neurobasal medium (Invitrogen, Carlsbad, CA) supplemented with B27 (Invitrogen) and L-glutamine (Invitrogen). Cells were then plated on poly-ornithin-coated (Sigma, St. Louis, MO; 10 µg/ml) 96-well white-walled optical plates (Nunc, Rochester, NY) at 100000 cells/well. Cultures were then incubated for 5 d *in vitro* (DIV) at 37°C and 7% CO_2_ before the neurotoxicity assay was started. For the assay recombinant Aβ_1-42_ (preparation see above) and 22C4 scFv were added directly to the cultures each at a final concentration of 5 µM and 0.5 µM, or were preincubated together for 4 h at 37°C and 500 rpm shaking before being added to the cells. Controls were treated with Ultra-Pure H_2_O for Cell Culture (Invitrogen). The plates were incubated for 48 h before evaluating neurotoxicity with the CytoTox-Glo™ Cytotoxicity assay (Promega, Madison, WI). The assay was performed according to the manufacturer's protocol. The luminescence was measured with a SpectraMax GeminiXS plate reader (Molecular Devices, Sunnyvale, CA). Each treatment group was measured in 12 replicates and each experiment was repeated 3 times. Data were calculated as means ± SD.

### Immunohistochemistry and Immunocytochemistry

Five micron paraffin sections of human AD and mouse brain tissue from APPswe/PS1dE9 [42], APPswe/PS1 [43] and ArcAβ [44] mice were mounted on glass slides and immunohistochemistry/immuno-fluorescence was performed; for DAB stainings the Vector Elite ABC kits (Vector Laboratories, Burlingham, CA) were used. The following antibodies were used for neuropathological analyses: 6E10 (1∶1000; Covance), anti-GFAP (1∶400; Advanced Immunochemicals, Long Beach, CA), 22C4 scFv (1∶100), 22C4 IgG (1∶500), anti-His (1∶100; Abcam, Cambridge, UK), anti-mouse biotinylated antibody (1∶500; Jackson, Suffolk, UK), anti-guinea pig Cy2 (1∶100; Jackson), anti-mouse Cy3 (1∶1000; Jackson) and anti-rabbit Cy3 (1∶1000; Jackson). Thioflavin S staining for fibrillar Aβ and CAA was performed by incubating slides in 0.25% KMnO_4_ for 20 min, followed by incubation in 2% K_2_S_2_O_5_/1% oxalic acid for 2 min, 0.25% acetic acid for 10 s and finally in 0.0125% Thioflavin S in 50% ethanol for 3 to 5 min followed by rinses in 50% ethanol and finally in distilled water. The Perl's Prussian blue method was used to visualize ferric iron in hemosiderin as a measure for microhemorrhages and was performed as published previously. For quantification of immunoreactivity, acquisition of images was performed using the DotSlide Virtual Slide System (Olympus, Tokyo, Japan). Acquisition time was held constant for all images. Computer-assisted analysis of images was performed using the Image J software. Plaque and CAA number was calculated for the cortex area in 5 equidistant saggital sections of all treated animals.

For Immunocytochemistry rat primary cortical neurons were prepared, cultured and treated according to the protocol for Cell Culture Toxicity Studies. After treatment the cells were washed with PBS and fixed with 4% PFA in PBS. For immunofluorescence the following antibodies were used: anti-Map2 (1∶1000; Millipore, Billerica, MA), anti- Aβ_1-42_ (1∶250; Calbiochem, San Diego, CA), anti-His (1∶1000, Abcam) and anti-Cleaved Caspase 3 (1∶100; Cell Signaling Technologies, Danvers, MA). The staining was repeated three times.

### Animals and Intranasal Treatment

Intranasal treatment was performed in 7.5 month-old male and female APPswe/PS1dE9 mice [42] on a C57BL/6 background, which were originally obtained from the Jackson Labs (Bar Harbour, ME). Animals were treated for 14 weeks starting at the age of 7.5 months, each of the 3 groups contained 15 animals; all groups were balanced for age gender. Control animals (treated with PBS, sterile, pH 7.4) and 22C4 IgG-treated animals received weekly intranasal applications (IgG concentration: 1 mg/ml). The 22C4 scFv-treated group was treated twice a week (scFv concentration: 1.5 mg/ml). With every application, animals received drop wise 20 µl of PBS, 22C4 IgG or 22C4 scFv by alternating between the nares. The application was performed in awake mice as described by Hanson et al. [45]. Briefly, the animals were grabbed by the skin of their necks and held firmly in the palm of the hand to minimize movement. During the total application time of 5 min each mouse was held in the hand in a supine position. After the treatment animals were returned to their home cage. All animal use was approved by the Swiss Institute for Animal Welfare and was in compliance with all federal and state regulations (approval ID 202-2005, Kantonales Veterinäramt Zürich).

After the final treatment animals received a lethal dose of Ketamine/Xylazine and were transcardially perfused with ice-cold PBS (pH 7.4). Brains were isolated and divided sagitally; one hemi brain was snap frozen in liquid nitrogen and stored at −80°C until further processing, the second hemisphere was post-fixed in 4% PFA in PBS, dehydrated and embedded in paraffin for histological analyses.

### Aβ-extraction from mouse brains and Aβ ELISA

The frozen brain halves of the intranasally treated animals were homogenized according to the protocol published by Shankar et al [46]. The resulting extracts TBS, TBS-Tx and GuHCl were stored at −80°C until further processing.

The Aβ_1-40_ and Aβ_1-42_ –levels in the resulting extracts were measured with the hAmyloid β40 and β42 ELISA Kits (The Genetics Company, Schlieren, Switzerland). ELISA was performed according to the manufacturer's protocol. Briefly, the standard and samples (in corresponding dilutions) were incubated together with an antibody conjugate o/n at 4°C on a plate pre-coated with antibody. After washing, the plates were incubated with enzyme conjugate for 30 min at RT. After incubation with the enzyme substrate for 25 to 30 min, the reaction was stopped by adding 1M H_2_SO_4_ and the plates were measured at 450 nm and 620 nm on a Tecan Sunrise plate reader (Tecan).

### Western Blot analysis

Brain extracts were analyzed by Western Blotting. Equal amounts of protein were separated on a 4–12% Bis-Tris gel (Invitrogen), transferred to a nitrocellulose membrane and blocked in TBS containing 5% skim milk and 0.1% Tween 20. Primary antibodies anti-His, 1∶500 (Qiagen) and 6E10, 1∶1000 (Covance) were incubated overnight at 4°C, and the corresponding secondary antibodies were incubated for 1 h at room temperature. SuperSignal West Dura (Pierce) was used for the detection of chemiluminescence. Signals were detected using the ImageQuant LAS4000 (GE Healthcare) and densitometric analysis was performed using the GeneTools software (Syngene, Frederick, MD) using the brain extracts from the vehicle cohort as reference for normalization.

### Statistical analysis

Data were expressed as means ± SEM. Statistical analysis was performed by ANOVA and Bonferronís or Fisheŕs PLSD Post-hoc tests using the GraphPad Prism 5 (GraphPad Software Inc., La Jolla, CA) and the StatView (SAS Institute Inc., Cary, NC) Software. A value of p<0.05 was considered statistically significant.

## Results

### Structure and binding properties of 22C4 scFv

**Figure 1:**
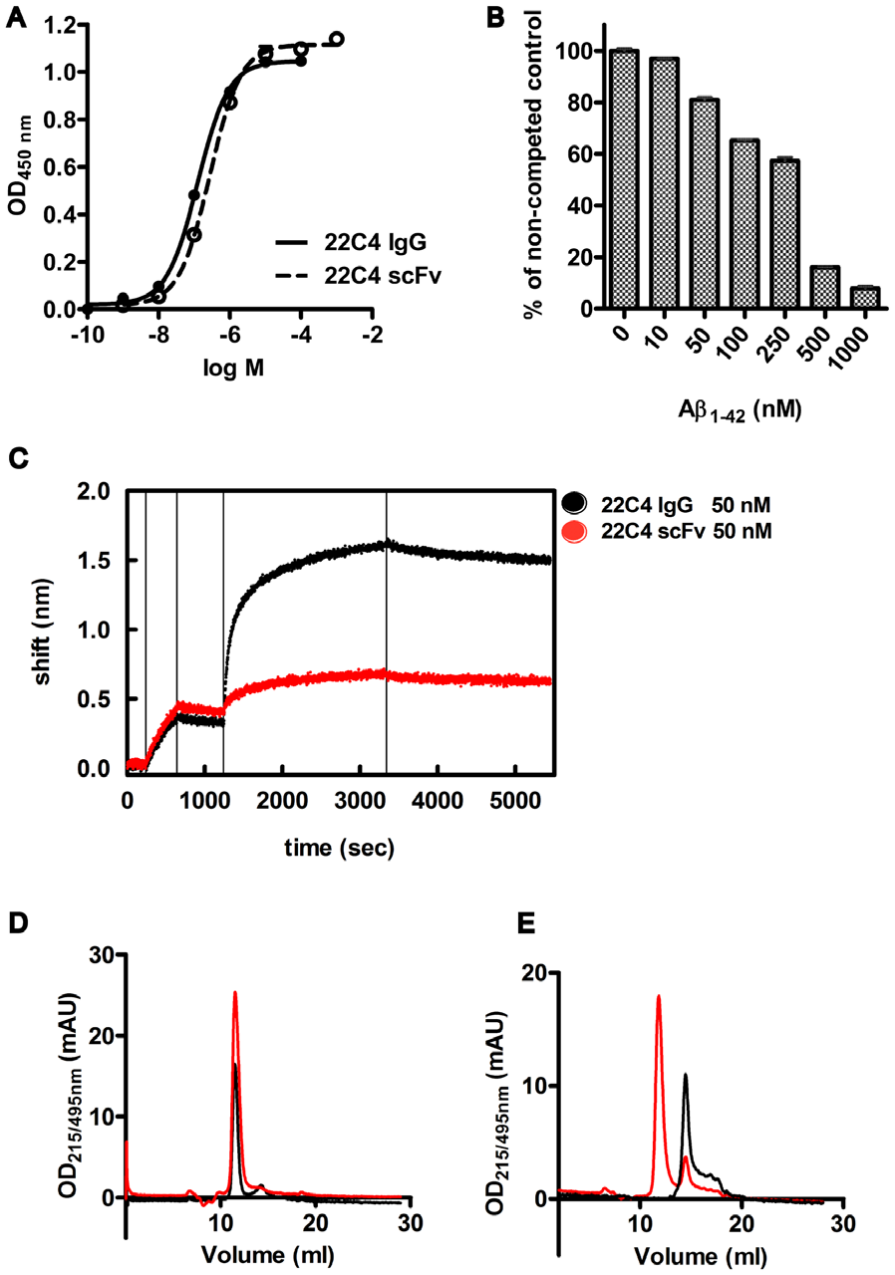
Binding properties of 22C4 scFv. **A** Binding activity of 22C4 scFv was determined by ELISA on an Aβ_1-42_-coated plate and compared to the corresponding full IgG 22C4. EC50 of 22C4 scFv was calculated to be 2.8*10^−7^ M and 1.3*10^−7^ M for 22C4 IgG. There was no difference between synthetic and recombinant Aβ_1-42_ as epitope on plate. **B** The specificity of 22C4 scFv to Aβ_1-42_ was determined by competition ELISA on Aβ_1-42_ - coated plate. 10 nM 22C4 scFv were competed with increasing amounts of recombinant Aβ_1-42_. Competition with scrambled Aβ_1-42_ did not show any non-specific binding to 22C4 scFv (Data not shown). **C** Determination of the K_D_ value by BioLayer Interferometry (Octet System). Both the scFv and the IgG were measured at 50 nM. 22C4 scFv was additionally measured at 75 and 125 nM (data not shown). The K_D_ value for 22C4 scFv was calculated to be 2.7*10^−8^ M and 1.2*10^−8^ M for 22C4 IgG. The shift of the curves between 22C4 IgG and scFv does not correlate with their affinity to Aβ_1-42_, but with the size of the molecule. **D and E** SEC analysis of FITC- Aβ_1-42_ (black, 495 nm) pre-incubated with either 22C4 scFv (red, 215 nm) or the non-specific scFv Fw 2.3 (red). 22C4 scFv and Aβ_1-42_ eluted together in a single peak (D) whereas Fw 2.3 and Aβ_1-42_ eluted in 2 different peaks (E).

The single-chain variable Fragment (scFv) antibody 22C4 scFv was generated by grafting the CDRs of the mouse monoclonal IgG 22C4 into a human scFv framework. The resulting antibody fragment (3D structure see Figure S1A) had a size of 26517 Da as determined by electrospray mass spectroscopy, was stable up to a temperature of 62.8°C as measured by FT-IR (Figure S1B) and retained the specificity of the full IgG for the amino acid residues 32–42 at the C-terminal end of the monomeric species of the amyloid-beta peptide (Fig. 1A). The EC50 of 22C4 scFv was calculated to be 280 nM and 130 nM for 22C4 IgG. However, there was no difference between synthetic and recombinant Aβ_1-42_ as epitope on plate (data not shown). As a more quantitative affinity assessment of the antibody-Aβ-complex, the K_D_ values of 22C4 scFv and 22C4 IgG were determined by BioLayer Interferometry (Octet System), in which the interference pattern of white light is measured. From the shift in the interference pattern the K_D_ value for 22C4 scFv was calculated to be 54 nM and 12 nM for 22C4 IgG (Fig. 1C). This difference in the apparent surface affinities is most probably due to the bivalent binding mode of the IgG as compared to the monovalency of the scFv.

To further characterize the specificity of 22C4 scFv binding to Aβ_1-42_ a competition ELISA and an analytical SEC were performed. For the competition ELISA 10 nM of 22C4 scFv were competed with increasing amounts of recombinant Aβ_1-42_. Accordingly, the signal of scFv bound to Aβ_1-42_ on the plate showed a dose-dependent decrease indicating the specificity of 22C4 scFv for Aβ_1-42_ (Fig. 1B). This was further substantiated as the competition with scrambled Aβ_1-42_ did not show any non-specific binding to 22C4 scFv (data not shown). During competition, the binding equilibrium of the scFv to its target peptide was not disturbed as the control plate showed 95% signal as compared to the assay plate. For the analytical SEC, FITC-labeled Aβ_1-42_ and either 22C4 scFv or the non-specific Fw2.3 were pre-incubated at equimolar concentrations (1 µM). The gel filtration revealed that 22C4 scFv and Aβ_1-42_ eluted together (Fig. 1D) whereas the non-specific control scFv Fw 2.3 and Aβ_1-42_ eluted at different volumes (Fig. 1E). The pre-incubation was repeated under different conditions varying incubation time and temperature, which both had no influence on the result (data not shown). FITC-Aβ_1-42_ and 22C4 scFv eluted together even when both were combined directly before injection into the column, indicating that binding of Aβ_1-42_ by 22C4 scFv was a fast and nearly complete reaction. These results suggest that the scFv 22C4 scFv is a small and highly specific antibody fragment that retained most of the binding properties of the full IgG 22C4 it was derived from. It exhibited good binding characteristics that are comparable to those of 22C4 IgG.

### 22C4 scFv binds to amyloid plaques both *in vitro* and *in vivo*

**Figure 2:**
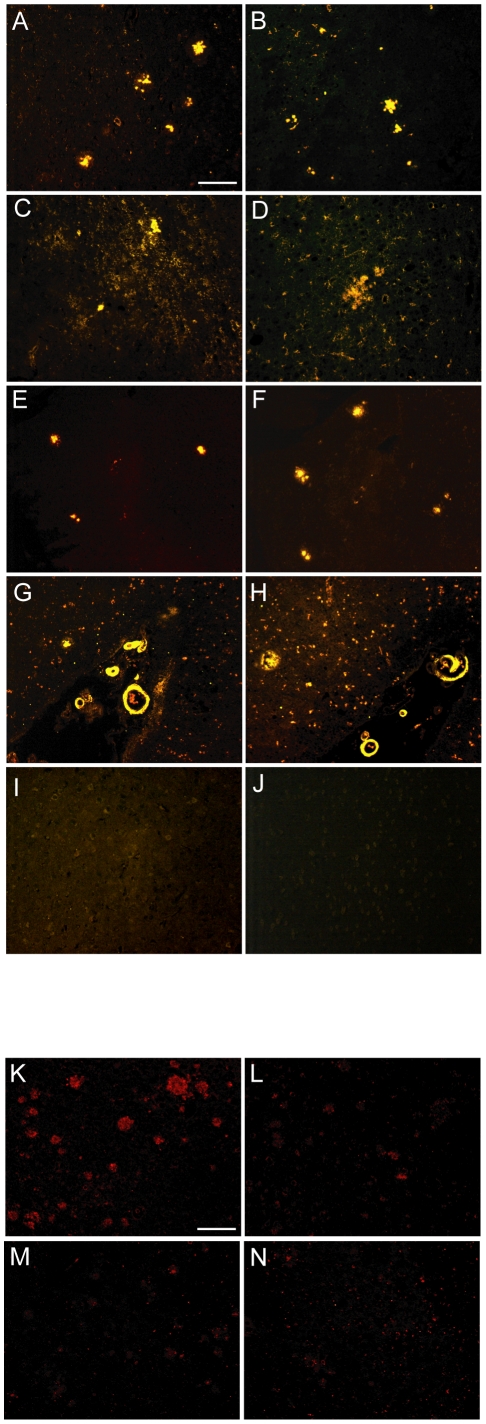
22C4 scFv binds to plaques both *ex* and *in vivo*. **A to J** Both the scFv and the corresponding full IgG were tested for their ability to label plaques in brains of different mouse models of AD and on human AD brains. Paraffin sections were stained with 22C4 scFv (A, C, E, G, I) and detected with an anti-His and an anti-rabbit Cy3 antibody or stained with 22C4 IgG (B, D, F, H, J), which was detected directly with an anti-mouse Cy3 antibody. APPswe/PS1 (A and B); ArcAbeta (C and D), APPswe/PS1dE9 (E an F), human AD (G and H), WT (I and J). Both the scFv and the full IgG recognized plaques in all tested tissues in comparable quality (Scale Bar: 200 µm). **K to N** After 12 weeks of intranasal application of 22C4 scFv (K) and 22C4 (M) in APPswe/PS1dE9 mice only the scFv was detectable in treated animals indicating that 22C4 IgG did not reach the brain in detectable amounts. The control animals were treated with PBS and stained for 22C4 scFv (L) and 22C4 IgG (N) accordingly (Scale Bar: 200 µm).

To test whether 22C4 scFv bound to Aβ_1-42_ not only in ELISA assays but also retained its activity on *ex vivo* tissue samples, brains from different AD mouse models and human AD brains were incubated with either 22C4 scFv (Fig. 2 A, C, E, G, I) or 22C4 IgG (Fig. 2 B, D, F, H, J). Both the scFv and the full IgG recognized amyloid plaques and CAA with comparable quality, but there were some differences between the tested tissues depending on the tissue origin. Amyloid deposits were best detected in APPswe/PS1 and APPswe/PS1dE9 brains with the least background staining (Fig. 2A and B, E and F). Deposits in human AD brains were detected with comparable sensitivity but with a somewhat stronger background staining (Fig. 2 G and H). Finally, both 22C4 scFv and 22C4 IgG were tested on sections from ArcAβ mice, which yielded the weakest staining of amyloid deposits with the strongest background staining (Fig. 2 C and D). Therefore, we decided to perform all following experiments in APPswe/PS1dE9 mice.

To test whether 22C4 IgG and 22C4 scFv also bound to amyloid plaques and CAA *in vivo*, brain sections from animals that were treated intranasally for 14 weeks with either 22C4 scFv (30 µg twice a week), 22C4 IgG (20 µg once a week) or PBS (once a week) were stained with an anti-5xHis antibody or an anti-mouse Cy3 antibody to detect 22C4 scFv and 22C4 IgG, respectively (Fig 2 K to N). The scFv clearly labeled plaques (Fig. 2 K), whereas the full IgG could not be detected in the brains of treated animals (Fig. 2 M). Control animals treated with PBS that were stained equally showed a weak background staining for both treatment groups (Fig. 2 L and N), but the staining for 22C4 scFv was much stronger as compared to the negative control. These suggested that scFv 22C4 had readily entered the brain and labeled amyloid plaques *in vivo*.

### 22C4 scFv inhibits both Aβ_1-42_ aggregation and neurotoxicity *in vitro* but does not bind to full-length APP

**Figure 3:**
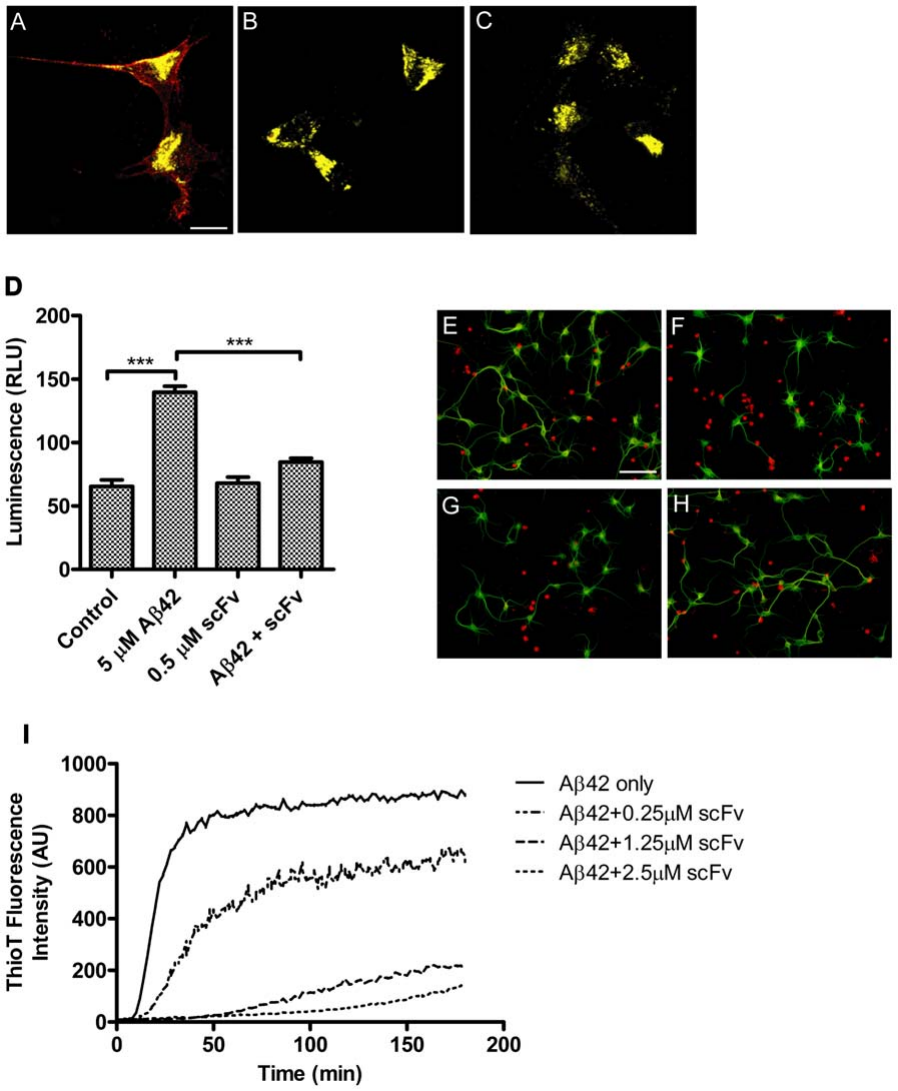
22C4 scFv inhibits Aβ_1-42_ aggregation and neurotoxicity in vitro without binding to full length APP. **A to C** HEK293T cells were transfected with an APP-Citrine (yellow) expressing construct. The cells were then incubated with either 6E10 (A), 22C4 scFv (B) or 22C4 IgG (C) (red) for 30 min on ice, fixed and fluorescence stained. The staining revealed that only 6E10 bound to full length APP. Incubation with control antibodies (Fw2.3, 2H6C2) showed similar staining compared to (B) and (C). Staining with secondary antibody did not show any staining (data not shown) (Scale Bar: 10 µm) **D to H** Primary Cortical Neurons from Rat (DIV5) were incubated with 5 µM recombinant Aβ_1-42_, 0.5 µM 22C4 scFv or both for 48 h. **D** Neurotoxicity analysis determined by Luminescence indicating cell death. Wells treated with Aβ_1-42_ showed significantly more dead cells as compared to control wells that were treated with H_2_O. The addition of 22C4 scFv significantly decreased cell death indicating that it inhibited Aβ_1-42_ neurotoxicity (*** p<0.005). **E to H** After treatment the cells were fixed with PFA and stained with anti-Map2 (green) and anti-Cleaved Caspase 3 (red) antibodies. Neurons in control wells (vehicle control E and 22C4 scFv G) and wells that were treated with Aβ_1-42_ and 22C4 scFv (H) showed better neuronal morphology with longer processes and less apoptotic neurons than neurons that were treated with 5 µM Aβ_1-42_ alone (F) (Scale Bar: 50 µm). **I** Monomeric recombinant Aβ_1-42_ (2.5 µM) was incubated at 25°C and 500 mM NaCl either alone or with 2.5 µM, 1.25 µM or 0.25 µM of 22C4 scFv. The Thioflavin T fluorescence of all four reactions was measured simultaneously every 3 min for 180 min. The addition of 22C4 scFv to the aggregation assay showed a dose-dependent effect on Aβ_1-42_ aggregation. Aggregation was delayed when using Aβ_1-42_-scFv ratios of 10∶1 and could almost be completely prevented when using equimolar concentrations.

Antibodies and antibody fragments directed against the amyloid beta-peptide could have the undesirable side effect to also bind to full-length APP, thereby possibly interfering with APP function and processing. To exclude this possibility we performed an *in vitro* binding assay in which HEK293T cells transfected with an APP-Citrine expressing construct were incubated with either antibody 6E10 as a positive control (Fig. 3 A), 22C4 IgG (Fig. 3 B), 22C4 scFv (Fig. 3 C) or corresponding non-specific antibodies for 30 min on ice (data not shown). After fixation fluorescence staining revealed that neither 22C4 scFv nor 22C4 IgG nor the non-specific control antibodies had bound to full-length APP, whereas 6E10 showed the expected co-localization (Fig. 3A). This finding confirmed that neither the scFv nor the full IgG specific for the C-terminus of Aβ_1-42_ bound to full-length APP suggesting that treatment with 22C4 scFv or 22C4 IgG was unlikely to be associated with an altered processing or trafficking of the APP molecule as a result of antibody binding; moreover, the finding excluded the possibility that full-length APP might act as a sink for the applied antibody.

We next tested whether 22C4 scFv was able to inhibit Aβ_1-42_–mediated neurotoxicity. Primary cortical neurons from rat embryos (E18) were incubated with 5 µM recombinant Aβ_1-42_, 0.5 µM 22C4 scFv or both for 48 h (Fig. 3 D), and neurons treated with both Aβ_1-42_ and 22C4 scFv exhibited significantly less cell death (*** p<0.005) when compared to cells treated with Aβ_1-42_ alone, indicating that 22C4 scFv indeed inhibited Aβ_1-42_-mediated neurotoxicity *in vitro*. This result was further supported by immunostaining of cultured rat cortical neurons which had been treated with 5 µM Aβ_1-42_, 0.5 µM 22C4 scFv or both for 24 h. The staining revealed that neurons in control wells and wells that had been treated with Aβ_1-42_ and 22C4 scFv showed more intact neuronal morphology with longer processes and less apoptotic neurons than neurons that were treated with 5 µM Aβ_1-42_ alone (Fig. 3 E to H). Immunostaining with an anti-Aβ_1-42_ antibody revealed that neurons were surrounded by Aβ_1-42_ aggregates. Aβ_1-42_ aggregates could also be detected in close association with significantly shortened neuritic processes (Figure S2).

The ability of 22C4 scFv to inhibit Aβ_1-42_ aggregation was assessed in an aggregation inhibition assay. Monomeric Aβ_1-42_ (2.5 µM) was incubated either alone or with the addition of 22C4 scFv at 2.5 µM, 1.25 µM or 0.25 µM (Fig. 3 I). The Thioflavin T fluorescence, which shows fluorescence at λ = 480 nm in the presence of β-sheet structures, was measured over a time of 180 min. The fluorescence did not increase when Aβ_1-42_ was co-incubated with equimolar concentrations of 22C4 scFv when compared to the peptide alone, indicating that the scFv inhibited Aβ_1-42_ aggregation. When the scFv was added to the assay at substoichiometric concentrations it reduced fibril formation rather than completely inhibiting Aβ_1-42_ aggregation. Notably, 22C4 scFv only inhibited aggregation completely when added to Aβ_1-42_ at the beginning of the assay. When added after the onset of aggregation, 22C4 scFv did not inhibit aggregation but rather delayed progression (Figure S3A). This observation further supported the idea that 22C4 scFv predominantly bound to linear (monomeric) state of Aβ_1-42_, and that it inhibited its aggregation via interference with the initial seed formation.

### 22C4 scFv lowers plaque number and reduces insoluble Aβ levels in the brains of intranasally treated APP transgenic mice

**Figure 4:**
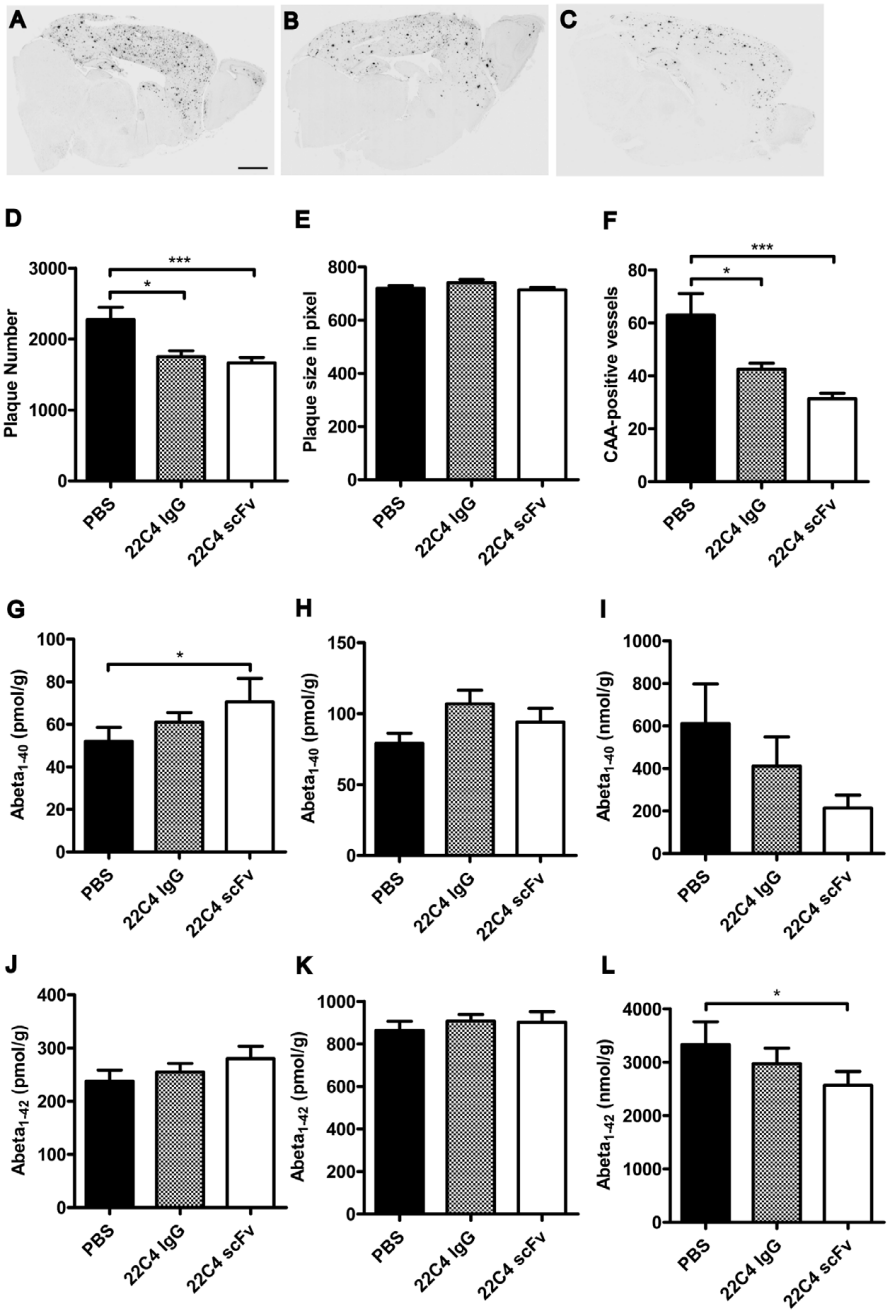
22C4 scFv reduced amyloid plaque number and influenced the amount of Aβ_1-40_ and Aβ_1-42_ in brains of treated animals. To determine the in vivo efficacy of 22C4 scFv APPswe/PS1dE9 mice were treated intranasally for 14 weeks with either 22C4 scFv, 22C4 IgG or PBS. After the final application animals were euthanized and brain sections were DAB stained with 6E10. **A to C** Representative pictures of PBS (**A**), 22C4 IgG (**B**) and 22C4 scFv (**C**) treated animals (Scale Bar: 1 mm). **D and E** Evaluation of 6E10 staining with Image J. The plaque number (D) was significantly reduced in 22C4 scFv and 22C4 IgG treated animals as compared to PBS treated animals, however, there was no difference in plaque size (**E**) between the treatment groups (* p<0.05). **F** Determination of CAA-positive vessels by Thioflavin S staining. The number of vessels with CAA was significantly decreased in mice treated with 22C4 IgG (* p<0.05) and 22C4 scFv (*** p<0.005) when compared to animals that had received PBS as a control. **G to I** Determination of Aβ_1-40_ and **J to L** Aβ_1-42_ amount in brain homogenates from intranasally treated APPswe/PS1dE9 mice by ELISA. In the TBS-fraction (**G and J**) the soluble Aβ_1-40_ was significantly increased in 22C4 scFv treated animals as compared to PBS treated animals, Aβ_1-42_ was slightly but not significantly increased in the 22C4 scFv treated group. Neither Aβ_1-40_ nor Aβ_1-42_ was significantly changed between the treatment groups in the membrane-bound TBS-Triton fraction (H and K). Both, the insoluble Aβ_1-40_ and Aβ_1-42_ in the GuHCl-fraction (I and L) were decreased. However, Aβ_1-40_ was only slightly reduced but Aβ_1-42_ was significantly decreased in the 22C4 scFv treated animals as compared to the PBS treated group (* p<0.05). Overall, we detected higher amounts of Aβ1-42 than Aβ1-40, which is in contrast to AD patients but correlates well with data published for the APPswe/PS1dE9 mouse model [42].

After determination of the binding characteristics and *in vitro* properties of 22C4 scFv, we further analyzed the scFv's *in vivo* efficacy. APPswe/PS1dE9 mice were treated intranasally for 14 weeks with either 22C4 scFv, 22C4 IgG or PBS, before significant amyloid deposition had occurred. After the final application, all brains were analyzed immunohistochemically and biochemically. The DAB staining of brain sections with 6E10 antibody showed a significant difference in plaque density between the treatment groups (Fig. 4 A to C). The quantitative analysis of the plaque number revealed that animals treated with 22C4 scFv had a significantly lower plaque number when compared to PBS treated animals (Fig 4 D; * p<0.05). However, there was no difference in plaque size between the treatment groups (Fig. 4 E). Additionally, Thioflavin S staining was performed to determine the number of CAA-positive vessels in the treated mice. This analysis showed a significantly decreased number of CAA-positive vessels in 22C4 scFv (*** p<0.005) and 22C4 IgG (* p<0.05) treated animals when compared to PBS treated controls (Fig. 4 F).

For further analysis hemi brains of all treated animals were homogenized and extraction of Aβ was performed. Aβ_1-40_ (Fig. 4 G to I) and Aβ_1-42_ levels (Fig. 4 J to L) in different fractions was then determined by ELISA. In the TBS fraction, soluble Aβ_1-40_ was significantly increased in the 22C4 scFv treated animals compared to PBS treated animals, whereas Aβ_1-42_ was slightly, though not significantly, increased. Neither Aβ_1-40_ nor Aβ_1-42_ levels were significantly different among the treatment groups in the membrane-bound TBS Triton fraction; in the “insoluble” GuHCl-fraction, however, levels of both Aβ_1-40_ and Aβ_1-42_ were significantly decreased. However, Aβ_1-40_ was only slightly reduced whereas Aβ_1-42_ was significantly decreased in the 22C4 scFv treated animals as compared to the PBS treated group (* p<0.05). Analysis of serum Aβ_1-40_ and Aβ_1-42_ levels revealed no statistically significant differences among the treatment groups (Figure S4). Moreover, Western blot analysis of TBS soluble extracts showed similar distributions of oligomeric Aβ species among the different treatment groups (Figure S5). The latter finding indicated that the increase of Aβ_1-40_ in the TBS fraction was not associated with an concomitant increase in potentially neurotoxic Aβ oligomers.

In summary, when administered intranasally, 22C4 scFv lowered the number of amyloid plaques and CAA-positive vessels in transgenic mice with brain amyloidosis. These results were further substantiated by the fact that the levels of Aβ_1-40_ and Aβ_1-42_ were significantly decreased in the insoluble fraction but increased in the soluble fraction of 22C4 scFv treated animals, suggesting a redistribution of brain Aβ from the insoluble fraction to the soluble peptide pool, which was not accompanied by an increase of serum Aβ levels or increased amounts of oligomeric Aβ species. Interestingly, all treatment effects were more pronounced in animals treated with scFv as compared to those treated with the corresponding full IgG.

## Discussion

Immunotherapeutic strategies for clearing Aβ from the brain [47] include active immunization by either systemic Aβ injection [5], [48], [49] or by nasal administration of the whole Aβ peptide [50], [51] or short Aβ immunogens [52], as well as passive immunization strategies with monoclonal antibodies [6], [15], [16], [53], [54], [55], [56], [57] or antibody fragments [29], [30]. Aβ immunotherapy can decrease both cerebral amyloid burden and brain levels of Aβ, attenuate neuritic dystrophy, astrogliosis, and behavioral deficits, restore blood-brain barrier integrity [54] and prevent synaptic degeneration [58].

In this study we generated a humanized scFv against the C-terminus of Aβ. It was derived from the full IgG 22C4 [56], [32] by grafting the CDRs into a human scFv framework. The resulting scFv antibody 22C4 scFv retained most of the binding properties of the full IgG 22C4 it had been derived from. The scFv exhibited good binding characteristics with high affinity and specificity to Aβ. 22C4 scFv was identified to bind to both synthetic and recombinant Aβ in *in vitro* assays as well as to natural Aβ in transgenic mouse and human tissues. Moreover, we showed that 22C4 scFv labeled Aβ deposits *in vivo*.

22C4 scFv potently inhibited Aβ aggregation and Aβ-mediated neurotoxicity *in vitro*, presumably by binding to Aβ monomers thereby blocking their assembly to larger and potentially neurotoxic oligomeric aggregates. Another possibility might be that this effect could be related to a sterical hindrance of Aβ to form aggregation seeds. Aggregation might be inhibited or disturbed when the concentration of an interfering agent is sufficient to modulate the formation of aggregation seeds as it is the case for human serum albumin (HSA) [59]. According to this theory, Rozga et al stated that the relative abundance of the low affinity HSA in serum is responsible for the aggregation-inhibiting effect on peripheral Aβ. In contrast, a specific Aβ-binding reagent should be able to inhibit aggregation at equimolar concentrations due to its higher affinity to Aβ [24]. Indeed, we could show that 22C4 scFv reduced Aβ aggregation even in substoichiometric concentration and it did so to a greater extent than the non-specific BSA (Figure S3B). Notably, 22C4 scFv only inhibited aggregation completely when added to Aβ_1-42_ at the beginning of the assay. When added after the onset of aggregation, 22C4 scFv could not inhibit aggregation but rather delayed aggregation progression. This observation further supports the idea that 22C4 scFv predominantly binds monomeric Aβ_1-42_, and that it can inhibit its aggregation as long as aggregation seeds have not yet formed. After the beginning of seed formation, aggregation can rather be delayed than inhibited. This might also explain how 22C4 scFv reduced Aβ-mediated neurotoxicity. After binding of the scFv to Aβ monomers, the formation of aggregation seeds and potentially toxic higher order species, such as oligomers and fibrils, was inhibited [47].

In this study we report the novel finding that chronic intranasal treatment of APPswe/PS1dE9 mice with 22C4 scFv and 22C4 IgG resulted in decreased Aβ accumulation in the parenchyma and vessels of the brain and accordingly also showed decreased Aβ levels in the insoluble fraction but increased levels in the soluble fraction of brain extracts. Animals treated with 22C4 scFv exhibited significantly less plaques in brain parenchyma, but there was no statistical difference in plaque size among the treatment groups. This might be due to the fact that the APP transgenic mice used in this study typically develop only small dense core plaques that do not grow significantly in size with disease progression [42]. As a result, the variation in plaque size is generally low in this model which might explain why our treatment lowered plaque numbers but did not significantly affect plaque size. The reduction in Aβ accumulation in brain parenchyma and brain vasculature was accompanied by a redistribution of Aβ from the insoluble to the soluble peptide pool. However, this redistribution was not accompanied by an increase in potentially neurotoxic Aβ oligomers as revealed by 6E10 Western Blotting (Figure S5).

Western blot analysis showed the presence of scFv in the brain as early as 1 h after intranasal application (Figure S6), suggesting rapid transport that is possibly compatible with the idea that 22C4 scFv enters the brain via perineural channels in the subarachnoid space of the nasal cavity and binds to amyloid plaques. The uptake of a molecule along the nasal pathway from nose to CNS is thought to involve two general mechanisms. The first is internalization of the molecule into the primary neurons of the olfactory epithelium and intracellular transport along the axon to the olfactory bulb and subsequent distribution in the brain tissue. The axonal route of transport is slow and molecules reach the CNS 24 h after application [36]. The second pathway, the olfactory epithelial pathway, has been reported to be faster [36]. It relies on a direct anatomic connection between the submucosa and the subarachnoid extensions, the perineural space surrounding the olfactory nerves, as they penetrate the cribiform plate [38]. It has been shown in rats that even large molecular weight compounds like nerve growth factor [60], insulin-like growth factor [33] and interferon-beta [35] can be transported rapidly into the CNS and distribute in the tissue in a time- and region-dependent manner. Accordingly, our results support the idea that 22C4 scFv was transported along the olfactory epithelial pathway, subsequently distributed in the brain tissue and was eliminated via blood [36], [38], [41]. After its entry and distribution in the CNS, there are three possible mechanisms by which 22C4 scFv might have exerted its effect on amyloid pathology: (i) by binding to monomers it could have prevented the formation of aggregation seeds and thus the accumulation of plaques, (ii) it interferes with plaque growth by binding to accessible C-terminal ends of the amyloid beta peptide or (iii) the binding of monomers might have reduced the concentration of free monomers which then could have led to the disintegration of fibrils in order to keep the equilibrium. Soluble monomers or scFv-bound monomers would then be subject to degradation or elimination from the brain by efflux mechanisms along the blood-brain barrier [47]. The latter “peripheral sink” mechanism, however, appears to be less likely responsible for the observed decrease in amyloid pathology in our study since we did not observe significant increases in serum Aβ levels in the 22C4 IgG- and scFv-treated animals as compared to the PBS-treated mice (Figure S4).

Most of the mechanisms proposed for the clearance of Aβ from the brain (for review see [47]) rely on the presence of therapeutically relevant antibody concentrations in the CNS. Antibody transport across the BBB into the brain thus is a critical issue. Available data from several studies estimate that only 0.1% to 1% of plasma antibodies enter the CNS [10], [11], [61]. Nevertheless, immunohistological analysis of brain tissue from participants of the AN1792 study showed antibodies bound to β-amyloid plaques [62]. In line with this study, we were also able to detect the scFv 22C4 scFv bound to plaques in the brains of treated APPswe/PS1dE9 mice. In the brain antibodies might be stabilized by binding to high local concentrations of target epitopes in β-amyloid plaques which possibly results in very slow apparent off-rates for the antibody-Aβ interaction. As a result, antibodies accumulate around brain Aβ deposits, and even small amounts of antibodies may thus be therapeutically relevant or cause therapeutically relevant antibody concentrations over time [1]. In order to increase the antibody concentration in the brain, we chose the intranasal route of delivery which circumvents the BBB and leads to a fast time- and region- dependent distribution of applied molecules in the brain [36], [38]. Studies using dextran [40] or NGF [63] in rats showed a strong dependence of the levels and the distribution of the applied biologics in brain parenchyma on molecular weight. These findings might also explain our observation why all our experimental treatment effects appeared to be more pronounced in mice treated with the smaller scFv as compared to the full IgG. It is possible that the full IgG either entered the brain in smaller quantities or that its distribution was strongly reduced due to its larger molecule size as compared to the scFv. Most studies that had evaluated intranasal application as route of delivery to the brain were performed in the rat model which most likely overestimates the degree of transport due to the relatively larger surface of the olfactory epithelium and relatively bigger olfactory bulbs of rats in comparison with humans [38], [39]. Some studies performed in humans, however, confirmed the existence of a direct pathway from the nose to the brain [38]. Born et al. [39] demonstrated that the intranasal delivery of melanocortin, vasopressin and insulin resulted in increased levels of these peptides in the CSF within 30 minutes, without increasing their levels in the bloodstream. Several studies also measured the pharmacological effects of intranasally delivered molecules, thereby strengthening the possibility of a direct and efficacious nose-to-brain transport also in humans [38].

Studies in transgenic mice indicated the possibility that antibody treatment leads to microhemorrhages associated with pre-existing CAA [15], [16]. This might be related to interactions of anti-Aβ antibodies with the vascular amyloid which causes local breaches of the BBB due to degenerated smooth muscle and endothelial cells [4], [17]. Antibody-mediated microhemorrhages can also be related to Fc effector functions since de-glycosylated IgG without effector functions show a significantly reduced risk to induce microhemorrhages [16], [54]. This might also be true for scFv since these antibody fragments miss the Fc effector domain, and in agreement with this, we did not detect significantly increased microhemorrhages in 22C4 scFv treated mice, when compared to PBS-treated animals (Figure S7). Another concern is that CAA can be increased due to antibody-mediated clearance of Aβ from the brain parenchyma and subsequent transport of Aβ to the vasculature and accumulation at the blood vessel walls [54], [62]. On the other hand, it was shown that anti-Aβ antibodies can also reduce vascular amyloid without increasing the incidence of microhemorrhages [64], [65]. It is possible that the propensity of an antibody to increase CAA is epitope-dependent [17]. The degree of treatment-related effects such as microhemorrhages might vary depending on the severity of baseline CAA pathology [61]. Furthermore, it was shown that an increased incidence of microhemorrhages in an immunization study could be controlled by lowering the antibody dosage [65]. Our results also demonstrated that the 22C4 scFv reduced CAA in transgenic mice without increasing microhemorrhages, which might be attributable to the route of delivery. As previously discussed Thorne et al. [33] states that one mechanism of intranasal delivery of protein therapeutics to the brain is via perivascular channels, which would put the scFv in direct contact with cerebrovascular Aβ. This is further supported by the fact that high levels of interferon β-1b [66] and hypocretin-1 [67] were detected in blood vessel walls after intranasal administration.

In this study we report for the first time that chronic intranasal treatment of transgenic mice before the onset of Aβ deposition with 22C4 scFv resulted in decreased Aβ accumulation in the parenchyma and vessels of the brain and accordingly also showed decreased Aβ levels in the insoluble fraction but increased levels in the soluble fraction. Our findings suggest that the scFv 22C4 may be a useful alternative to full IgG molecules for reducing brain Aβ without triggering Fc-mediated effector functions and, possibly, microhemorrhages. Furthermore, these findings broaden the therapeutic potential of anti-Aβ immunotherapy to the removal of beta-amyloid from CAA lesion. Finally, this study further supports intranasal delivery of protein therapeutics for the treatment of CNS disorders, which has previously been shown to be efficacious in non-human primates [68] and humans [69], [70].

## Supporting Information

Figure S1 **A** 3D-model of 22C4 scFv generated using the SWISS-MODEL Repository which is a database of annotated three-dimensional comparative protein structure models generated by the fully automated homology-modeling pipeline SWISS-MODEL. The repository was developed at the BioCenter Basel (Swiss Institute of Bioinformatics). Blue: linker; Red: CDRs; Green: site where 22C4 scFv is hydrolyzed. **B** Measurements of the FT-IR spectrum with increasing temperature from 25°C to 95°C to determine thermal stability. The melting temperature of 22C4 scFv (50% unfolding of the protein) was determined to be at 62.8°C.
(TIF)

Figure S2 Immunostainings of primary rat cortical neurons (DIV5) which had been incubated with 5 µM recombinant Aβ42 before they were fixed with paraformaldehyde and stained with anti-Map2 (green) and anti-Aβ_1-42_ (red) antibodies. Neurons show damaged neuronal morphology, swollen somata and shortened processes in close association with Aβ42 aggregates (Scale Bar: 50 µm).
(TIF)

Figure S3 **A** 2.5 µM monomeric recombinant Aβ42 was incubated in 500 mM NaCl at 25°C alone or with equimolar concentrations of 22C4 scFv which was added to the stirred assay either when ThioT fluorescence reached half-maximal or maximal intensity. Complete inhibition of Aβ42 aggregation by 22C4 scFv was only achieved when 22C4 scFv was added to Aβ42 at the beginning of the assay. When added after onset of aggregation, 22C4 scFv did not effectively inhibit Aβ42 aggregation but rather delayed the progression of the aggregation process. **B** Monomeric recombinant Aβ42 was incubated in 500 mM NaCl at 25°C alone or with either equimolar or half-equimolar concentrations of BSA. When Aβ42 was incubated with BSA that binds to Aβ42 unspecifically, only a slight reduction in fibril formation was detectable even with equimolar concentrations of BSA.
(TIF)

Figure S4 Serum Aβ_1-40_ (**A**) und Aβ_1-42_ (**B**) levels in the intranasally treated APPswe/PS1dE9 mice as determined by ELISA. Serum was taken at the beginning of the treatment period and after the application of the final dose of the biologic. Aβ levels at the beginning of the treatment were subtracted from the levels at the end of the treatment period. No significant differences in serum Aβ_1-40_ and Aβ_1-42_ levels could be detected among the different treatment groups, although 22C4 IgG and 22C4 scFv treated mice tended to show lower Aβ_1-40_ levels at the end of the intranasal treatment.
(TIF)

Figure S5 **A** Exemplary Western Blot of TBS brain extracts from intranasally treated APPswe/PS1dE9 mice. Bands detected with 6E10 antibody had apparent molecular weight of 4 kDa (corresponds to Aβ monomer), 14 kDa (trimer), 22 kDa (hexamer) and 52 kDa (probably corresponds to dodecamer). **B** Quantification of bands revealed no significant differences in oligomer distribution between treatment groups.
(TIF)

Figure S6 Western blot analysis of brain extracts from intranasally treated animals that were sacrificed 1 h after treatment with 200 µg 22C4 scFv. For the detection of the scFv, an anti-His primary antibody (Cell Signaling, 1∶1000) was used. It recognizes a 27 kDa band, which corresponds to the size of the scFv, and which could be detected in cortical (CX) and hippocampal (HPC) extracts. No significant amounts of scFv were detectable in the cerebellum (CBL) and the olfactory bulb (OB) after 1 h. 6 h after the treatment, scFv was not detectable by Western Blotting in any region (data not shown).
(TIF)

Figure S7 Perl's Prussian blue histological stainings for microhemorrhages (counterstained with Nuclear Fast Red) of paraffin sections from intranasally treated animals. Microhemorrhages were slightly but not significantly increased in 22C4 scFv treated animals when compared to control animals. Microhemorrhages were most abundant in the olfactory bulb. Representative pictures of PBS (**A**), 22C4 IgG (**B**) and 22C4 scFv treated animals (**C**) after 14 weeks of treatment (Scale bar: 200 µm). **D** Quantitative analysis of Perl's Prussian blue histological stainings revealed that microhemorrhages were slightly but not significantly increased in 22C4 scFv treated animals when compared to control animals.
(TIF)
