# Supplementary figures and images for: Chronic Intranasal Treatment with an Anti-Aβ30-42 scFv Antibody Ameliorates Amyloid Pathology in a Transgenic Mouse Model of Alzheimer's Disease
Source: PLoS One. 2011 Apr 5;6(4):e18296. doi: 10.1371/journal.pone.0018296 (PMC3071717; doi:10.1371/journal.pone.0018296)

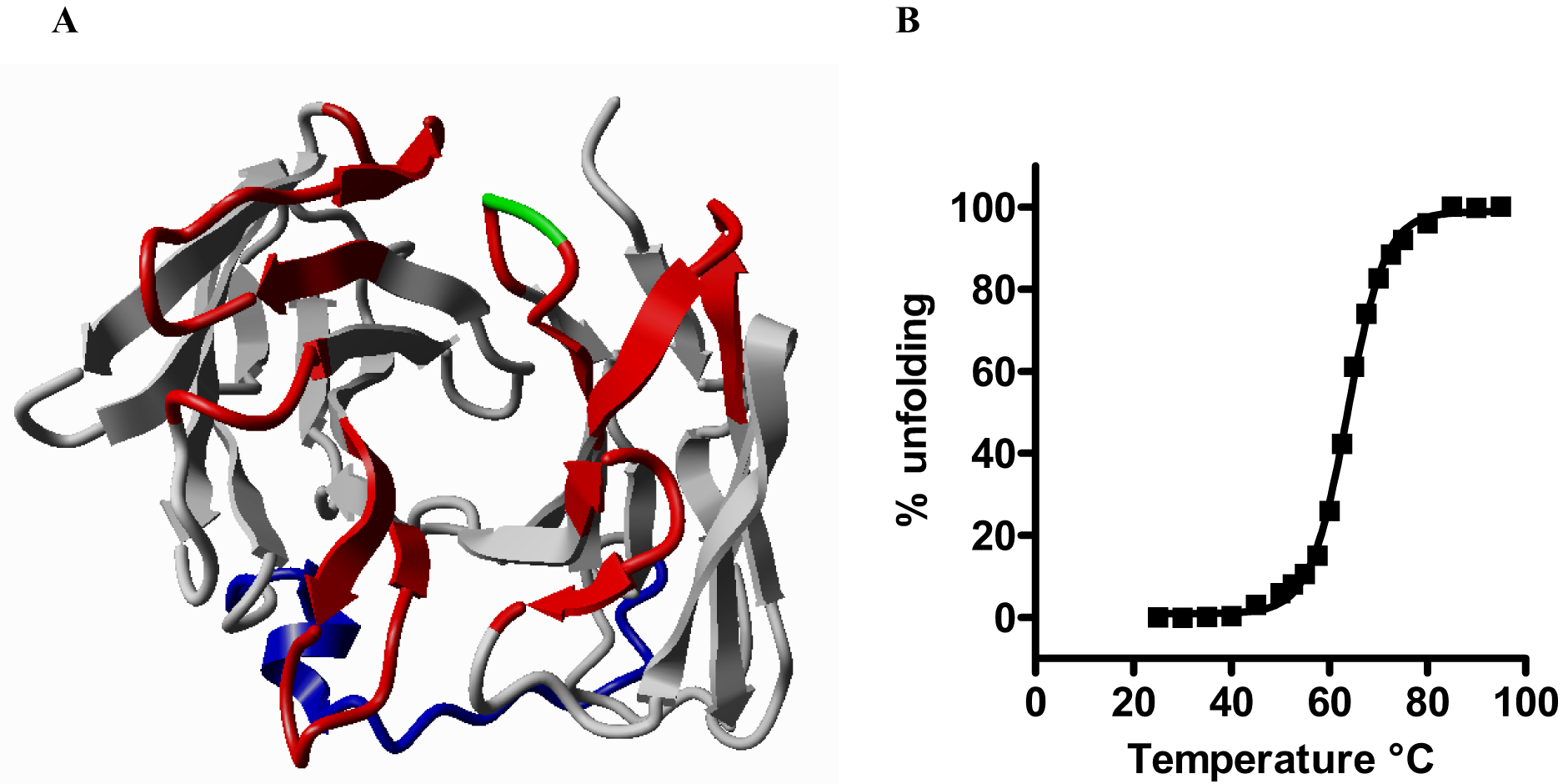

Supplement: Figure S1 — A 3D-model of 22C4 scFv generated using the SWISS-MODEL Repository which is a database of annotated three-dimensional comparative protein structure models generated by the fully automated homology-modeling pipeline SWISS-MODEL. The repository was developed at the BioCenter Basel (Swiss Institute of Bioinformatics). Blue: linker; Red: CDRs; Green: site where 22C4 scFv is hydrolyzed. B Measurements of the FT-IR spectrum with increasing temperature from 25°C to 95°C to determine thermal stability. The melting temperature of 22C4 scFv (50% unfolding of the protein) was determined to be at 62.8°C. (TIF) [file pone.0018296.s001.tif]

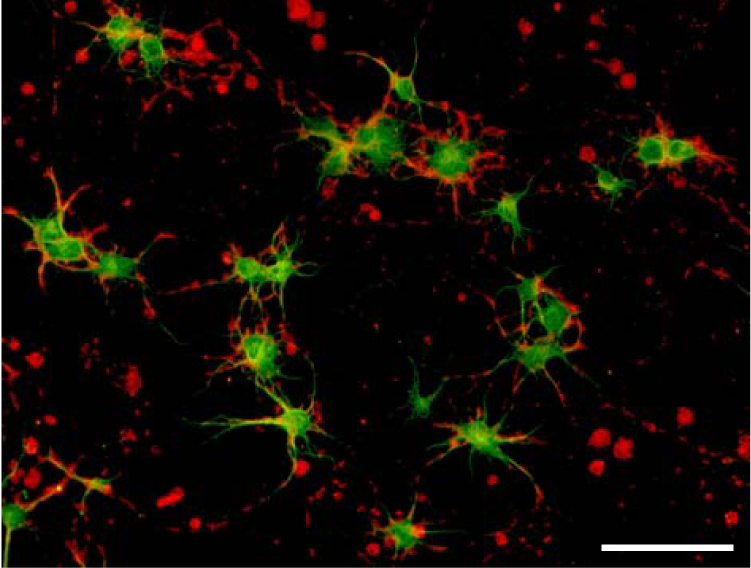

Supplement: Figure S2 — Immunostainings of primary rat cortical neurons (DIV5) which had been incubated with 5 µM recombinant Aβ42 before they were fixed with paraformaldehyde and stained with anti-Map2 (green) and anti-Aβ1-42 (red) antibodies. Neurons show damaged neuronal morphology, swollen somata and shortened processes in close association with Aβ42 aggregates (Scale Bar: 50 µm). (TIF) [file pone.0018296.s002.tif]

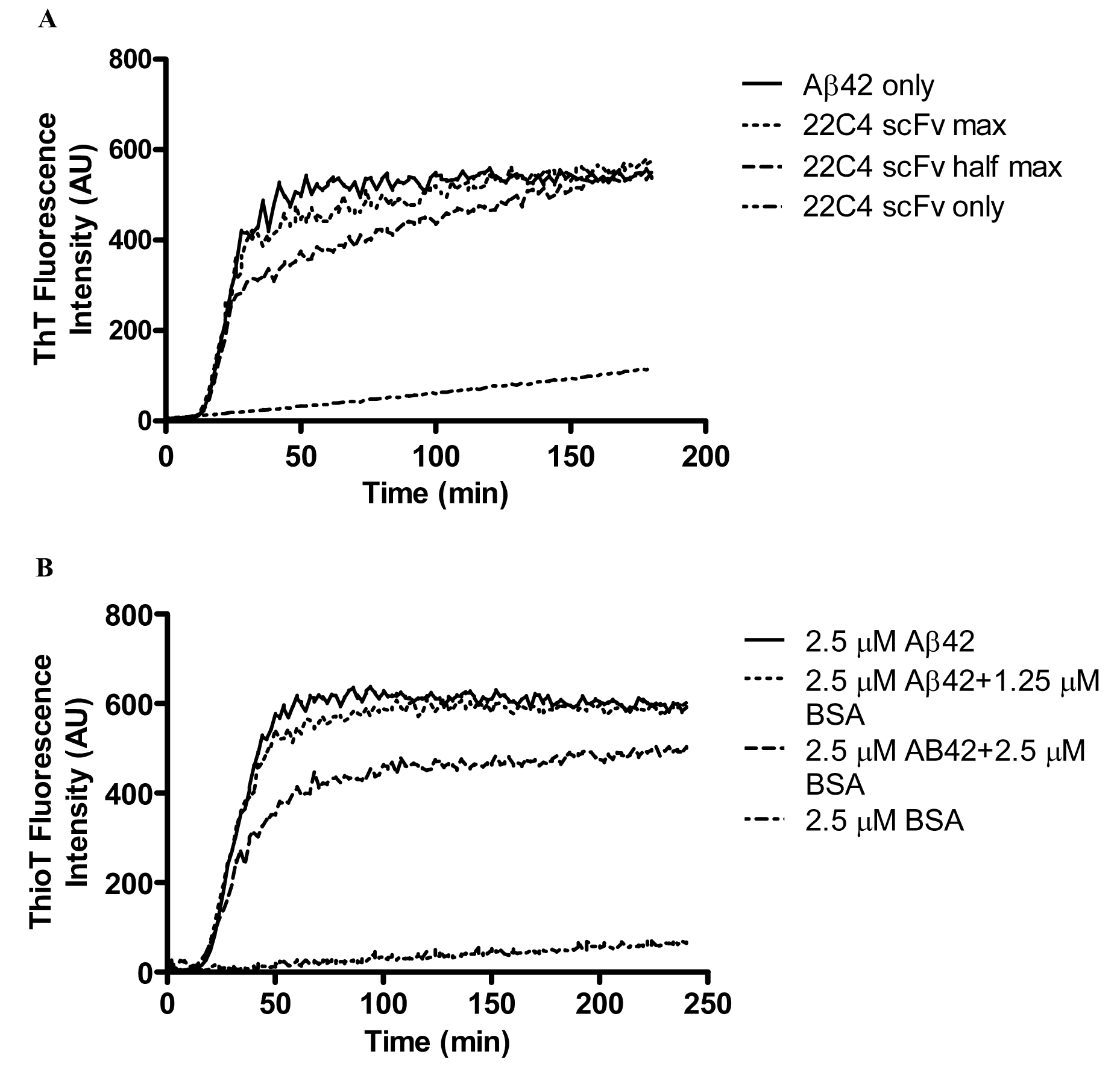

Supplement: Figure S3 — A 2.5 µM monomeric recombinant Aβ42 was incubated in 500 mM NaCl at 25°C alone or with equimolar concentrations of 22C4 scFv which was added to the stirred assay either when ThioT fluorescence reached half-maximal or maximal intensity. Complete inhibition of Aβ42 aggregation by 22C4 scFv was only achieved when 22C4 scFv was added to Aβ42 at the beginning of the assay. When added after onset of aggregation, 22C4 scFv did not effectively inhibit Aβ42 aggregation but rather delayed the progression of the aggregation process. B Monomeric recombinant Aβ42 was incubated in 500 mM NaCl at 25°C alone or with either equimolar or half-equimolar concentrations of BSA. When Aβ42 was incubated with BSA that binds to Aβ42 unspecifically, only a slight reduction in fibril formation was detectable even with equimolar concentrations of BSA. (TIF) [file pone.0018296.s003.tif]

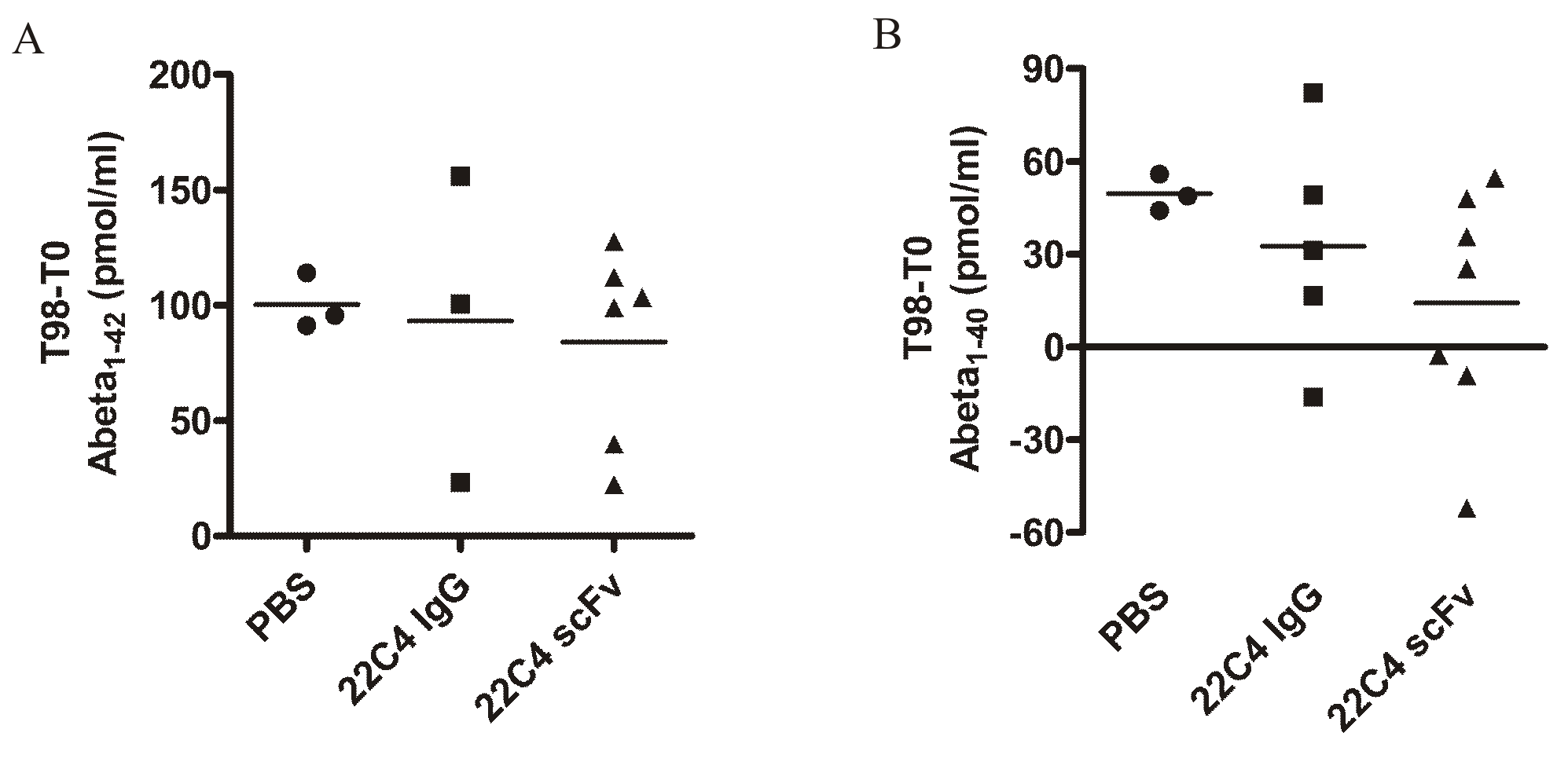

Supplement: Figure S4 — Serum Aβ1-40 (A) und Aβ1-42 (B) levels in the intranasally treated APPswe/PS1dE9 mice as determined by ELISA. Serum was taken at the beginning of the treatment period and after the application of the final dose of the biologic. Aβ levels at the beginning of the treatment were subtracted from the levels at the end of the treatment period. No significant differences in serum Aβ1-40 and Aβ1-42 levels could be detected among the different treatment groups, although 22C4 IgG and 22C4 scFv treated mice tended to show lower Aβ1-40 levels at the end of the intranasal treatment. (TIF) [file pone.0018296.s004.tif]

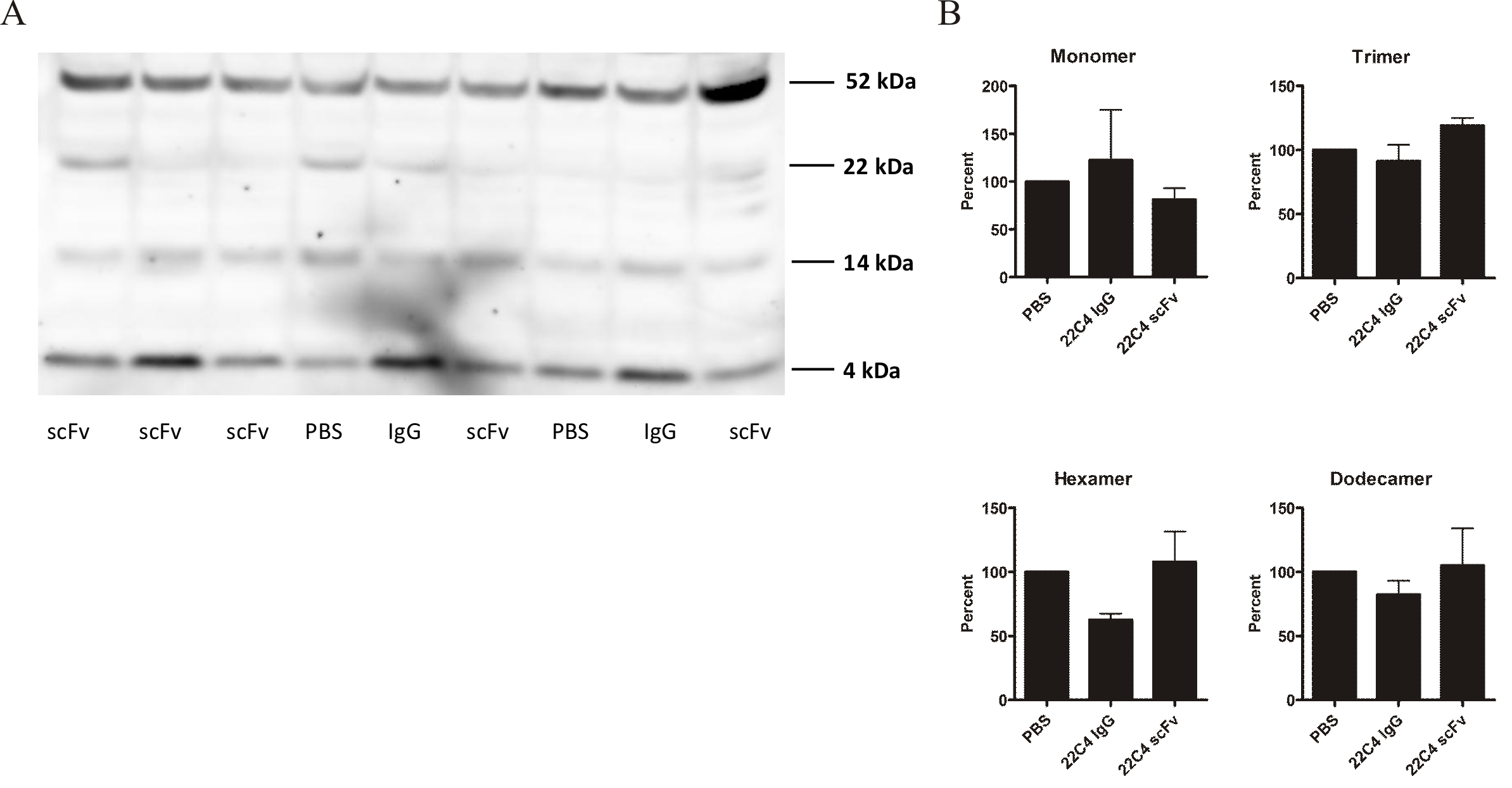

Supplement: Figure S5 — A Exemplary Western Blot of TBS brain extracts from intranasally treated APPswe/PS1dE9 mice. Bands detected with 6E10 antibody had apparent molecular weight of 4 kDa (corresponds to Aβ monomer), 14 kDa (trimer), 22 kDa (hexamer) and 52 kDa (probably corresponds to dodecamer). B Quantification of bands revealed no significant differences in oligomer distribution between treatment groups. (TIF) [file pone.0018296.s005.tif]

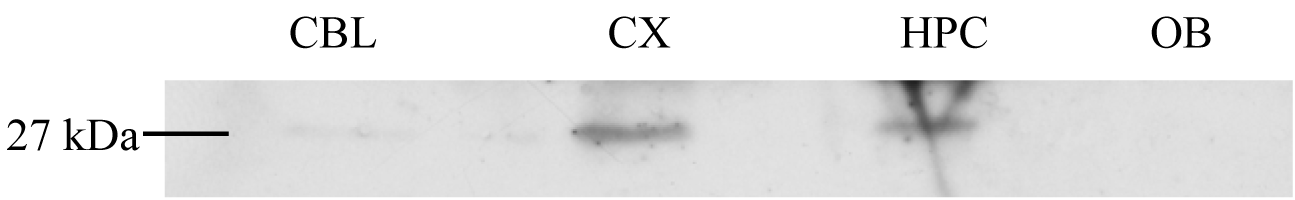

Supplement: Figure S6 — Western blot analysis of brain extracts from intranasally treated animals that were sacrificed 1 h after treatment with 200 µg 22C4 scFv. For the detection of the scFv, an anti-His primary antibody (Cell Signaling, 1∶1000) was used. It recognizes a 27 kDa band, which corresponds to the size of the scFv, and which could be detected in cortical (CX) and hippocampal (HPC) extracts. No significant amounts of scFv were detectable in the cerebellum (CBL) and the olfactory bulb (OB) after 1 h. 6 h after the treatment, scFv was not detectable by Western Blotting in any region (data not shown). (TIF) [file pone.0018296.s006.tif]

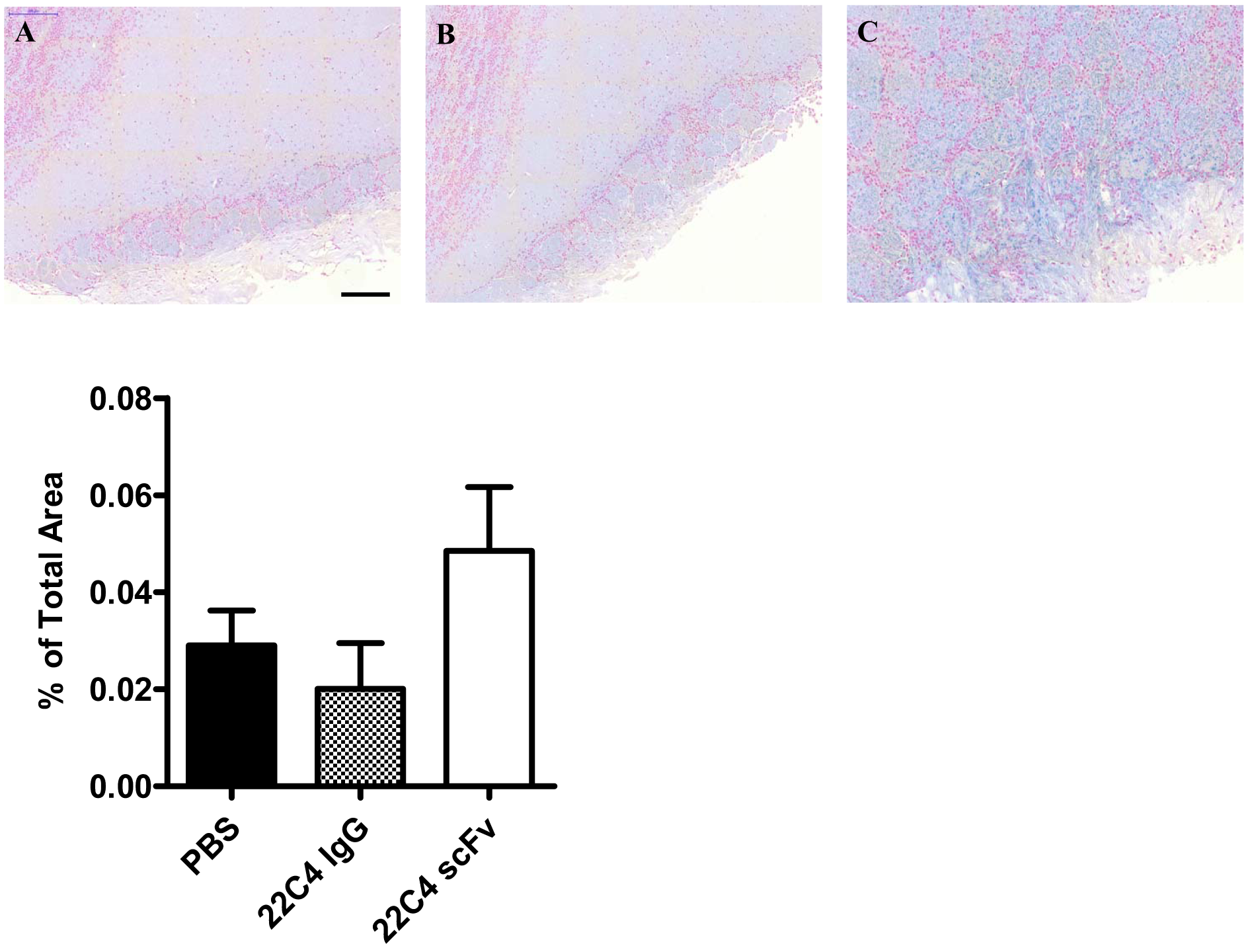

Supplement: Figure S7 — Perl's Prussian blue histological stainings for microhemorrhages (counterstained with Nuclear Fast Red) of paraffin sections from intranasally treated animals. Microhemorrhages were slightly but not significantly increased in 22C4 scFv treated animals when compared to control animals. Microhemorrhages were most abundant in the olfactory bulb. Representative pictures of PBS (A), 22C4 IgG (B) and 22C4 scFv treated animals (C) after 14 weeks of treatment (Scale bar: 200 µm). D Quantitative analysis of Perl's Prussian blue histological stainings revealed that microhemorrhages were slightly but not significantly increased in 22C4 scFv treated animals when compared to control animals. (TIF) [file pone.0018296.s007.tif]
